# Characterization of the complete plastid genome of *Gaultheria griffithiana* (Ericaceae)

**DOI:** 10.1080/23802359.2021.1914227

**Published:** 2021-04-30

**Authors:** Yi-Rong Li, Yan-Ling Xu, Xin-Yu Du, Shu-Da Yang, Lu Lu

**Affiliations:** a School of Pharmaceutical Sciences and Yunnan Key Laboratory of Pharmacology for Natural Products, Kunming Medical University, Kunming, China; b Plant Germplasm and Genomics Center, Germplasm Bank of Wild Species, Kunming Institute of Botany, Chinese Academy of Sciences, Kunming, China

**Keywords:** Chloroplast genome, Ericaceae, *Gaultheria griffithiana*, Leucothoides

## Abstract

*Gaultheria griffithiana* is an evergreen shrub in the family Ericaceae. It is used as a source of the Chinese traditional medicine, Tougucao, with distribution of the junction of eastern Himalaya and Hengduan Mountain. The chloroplast genome of *G. griffithiana* is 175,649 bp in length with 135 genes, including eight rRNA genes, 39 tRNA genes, and 85 protein-coding genes. Phylogenetic analysis has converged on the placement of *G. griffithiana* as sister to *G. praticola*, *G. nummularioides*, and *G. hookeri* within the Leucothoides clade of *Gaultheria* in this study.

*Gaultheria griffithiana* Wight belongs to the Leucothoides clade of *Gaultheria* of the tribe Gaultherieae within Ericaceae (Lu et al. 2019), which has been indigenously of use to treat rheumatism and anti-inflammatories due to containing benzoic acid derivatives, anthraquinones and alkaloids (Liu et al. 2013). As an evergreen shrub, *G. griffithiana* has a unique geographical distribution in the junction of eastern Himalaya and Hengduan Mountain including northeast India, Indochina (Myanmar, Vietnam), and southwest China, with elevations ranging from 2000 m to 3600 m (Fang and Stevens 2005; Fritsch et al. 2008). Based on the combined data of multiple genes from ITS and plastid loci, *G. griffithiana* was not monophyletic in the Leucothoides clade and its phylogenetic position was unresolved in the work of Lu et al. (2010). However, this species was subsequently resolved as a sister to most species such as *G. nummularioides* D. Don, *G. praticola* C. Y. Wu, and *G. hookeri* C. B. Clarke from the Leucothoides clade in the work of Fritsch et al. (2011). 

The plastid genome data has been proved to apparently well improve the phylogenetic resolution of species within *Gaultheria* series *Trichophyllae* from distinct subclade within the Leucothoides clade (Zhang et al. 2017). Herein, we characterized the complete plastid genome sequence of *G. griffithiana* using Illumina sequencing data, for the purposes to understanding the phylogenetic position of the species, further evolutionary and pharmacological studies.

The leaf sample of *G. griffithiana* was collected from Cangshan Mountain of Dali City (25°52′12″N, 100°1′12″E) in the southwest of Yunnan Province, China. The voucher specimen (number: KUN1248996) was deposited in the herbarium at Kunming Institute of Botany (KUN). Total genomic DNA was extracted with the CTAB (Cetyltrimethyl Ammonium Bromide) protocol (Doyle and Doyle 1987), followed by insert size of 150 bp paired-end sequencing using Illumina Hiseq X-Ten Sequencing System (the Molecular Biology Experiment Center, the Germplasm Bank of Wild Species in Southwest China) with standard Illumina sequencing protocols (Shendure and Ji 2008). The complete chloroplast genome was assembled using SPAdes v3.10.1 (Bankevich et al. 2012) and improved by the GetOrganelle pipeline (Jin et al. 2018) with *Vaccinium macrocarpon* Aiton (GenBank accession: NC019616.1) as reference, and then annotated using the Geneious R8 (Kearse et al. 2012). The phylogenetic tree of 19 species selected within Ericaceae was reconstructed based on complete chloroplast genome sequences which were downloaded from NCBI GenBank, with full-length sequences aligned by MAFFT v7 software (Katoh and Standley 2013). We used maximum likelihood analysis (option ‘-f a’) with 1000 rapid bootstrap replicates under GTRGAMMA model on Cipres Science Gateway (Miller et al. 2015, available at www.phylo.org).

The complete chloroplast genome sequence of *G. griffithiana* is 175,649 bp in length with a large single-copy region (LSC) of 107,114 bp, a small single-copy region (SSC) of 3,693 bp, and two inverted repeat regions (IRa and IRb) of 32,421 bp each. Furthermore, the genome encodes 135 genes including 85 protein-coding genes, 39 tRNAs, and eight rRNAs. The overall GC content of the chloroplast genome is 36.6%, and the corresponding GC values in LSC, SSC, and IR regions are 35.6%, 27.8% and 38.7%, respectively. Annotated chloroplast genome sequence was submitted to GenBank with an accession number MW528025. The phylogenetic analysis has converged on the placement of *G. griffithiana* as sister to *G. praticola*, *G. nummularioides*, and *G. hookeri* within the Leucothoides clade of *Gaultheria* in this study (Figure 1), which was consistent with the topology based on ML analysis of combined chloroplast, and chloroplast plus nuclear sequence data in Fritsch et al. (2011). This provides additional rationale for using chloroplast data when reconstructing species relationships in *Gaultheria*.

**Figure 1.**
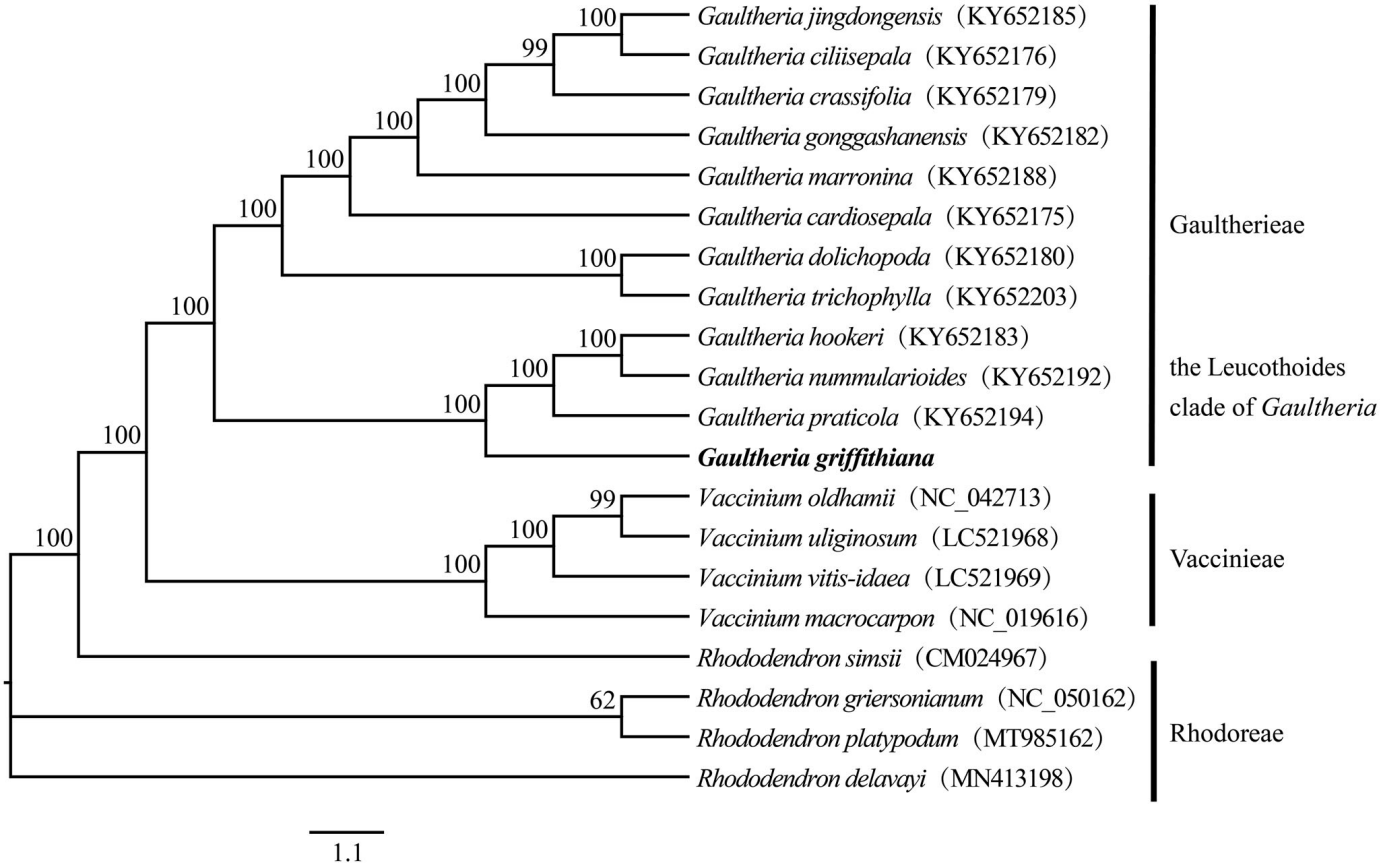
Phylogenetic tree based on 20 complete chloroplast genome sequences of Ericaceae. The bootstrap values are presented above each branch (only those > 50% are shown).

## Data Availability

The genome sequence data that support the findings of this study are openly available in GenBank of NCBI at (https://www.ncbi.nlm.nih.gov/) under the accession no. MW528025. The associated BioProject, SRA, and Bio-Sample numbers are PRJNA703716, SRX10145200, and SAMN18022285, respectively.
